# Supplementary material for: Comprehensive Annotation and Functional Exploration of MicroRNAs in Lettuce
Source: Front Plant Sci. 2021 Dec 24;12:781836. doi: 10.3389/fpls.2021.781836 (PMC8739914; doi:10.3389/fpls.2021.781836)
Supplement: Supplementary file 3 [file Data_Sheet_9.ZIP › GSM2230752.plot/Lsa-miRN46_Lsat_1_v5_gn_8_32760.1_9719_TPlot.pdf]

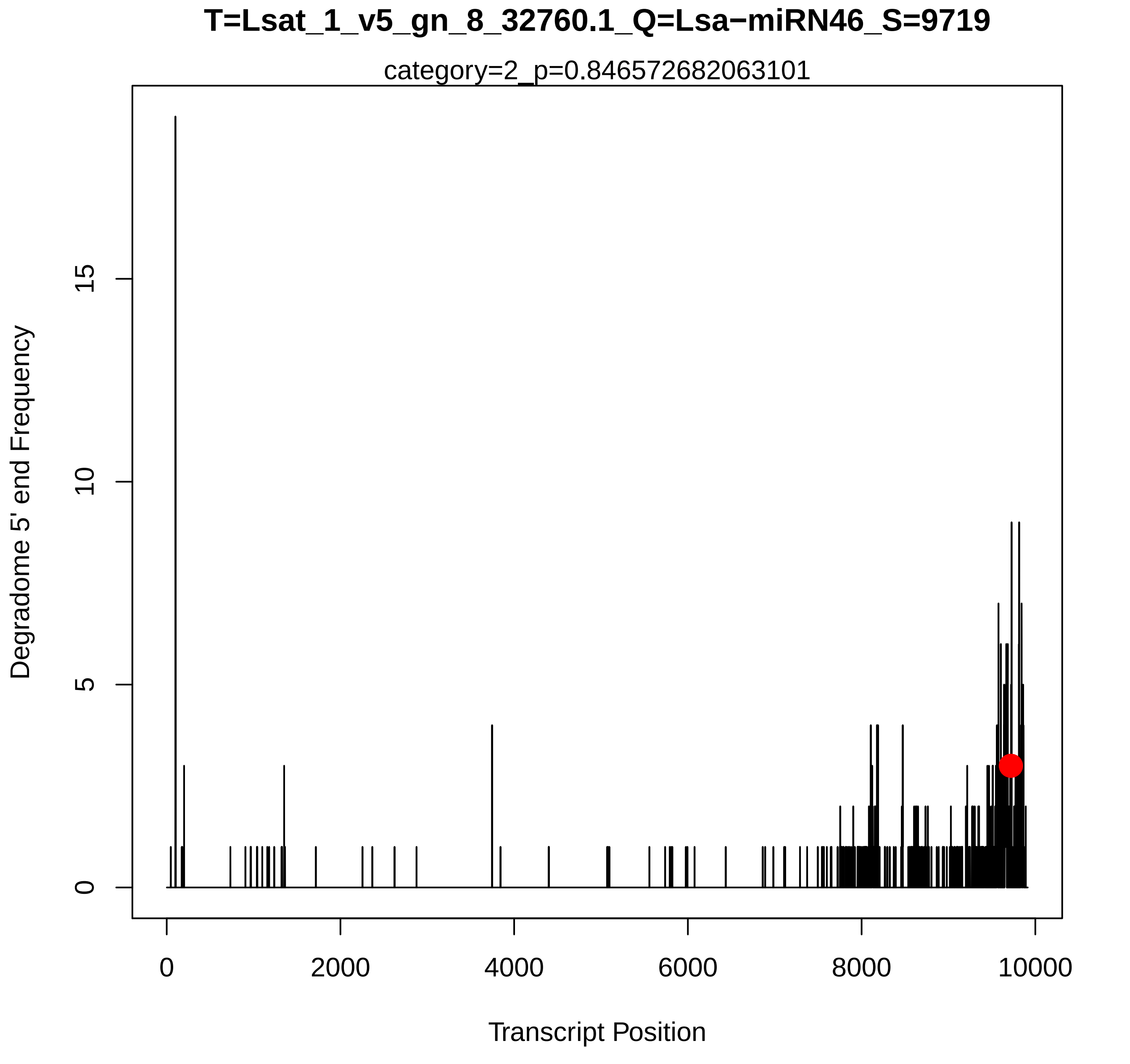

T=Lsat_1_v5_gn_8_32760.1_Q=Lsa−miRN46_S=9719
category=2_p=0.846572682063101
Degradome 5' end Frequency
0
5
10
15
0
2000
4000
6000
8000
10000
Transcript Position
